# Epithelioma and Varicose Ulcer

**Published:** 1885-11

**Authors:** Henry Wile

**Affiliations:** Lecturer on Skin Diseases in the Atlanta Medical College, Atlanta, Ga.


### Clinic Reports.

### EPITHELIOMA AND VARICOSE ULCER.

A CLINICAL LECTURE DELIVERED OCTOBER 10TH, BY HENRY WILE, M. D., LECTURER ON SKIN DISEASES IN THE ATLANTA MEDICAL COLLEGE, ATLANTA, GA.

Reported Stenographically for THE ATLANTA MEDICAL AND SURGICAL JOURNAL BY W. K. TEWKSBURY.

According to the statement of this man, who is fifty-six years of age, there appeared fourteen years ago a pin-head sized papule on the skin immediately outside of the inner canthus of the right eye towards the side of the nose. The top of this papule, after a few weeks, ulcerated, and the secretion drying formed a crust or scale. In four or six weeks this scale fell off, and after a short time another crust formed which continued about the same time and again dropped off. This process continued for several years, until finally a crust ceased forming as the secretion became more profuse. This secretion was of a viscid, yellowish nature. The ulceration extended towards the bridge of the nose and upwards to the border of the conjunctiva, forming an excavated dime-sized lesion with raised border and uneven surface. The patient then went under treatment, and, according to his statement, strong caustic remedies were applied. The result of the treatment was a healing up of the ulceration, with the exception of a small spot near the inner canthus of the eye. This lesion slowly continued to increase in size until he came under my notice, about one month ago, when the following appearances presented themselves : In the inner canthus of the right eye, extending upward towards the bridge of the nose and downwards toward the side of the nose, was an irregularly shaped dime-sized ulceration, with uneven, rough surface, and covered with a yellowish, jelly-like secretion. There was no pain attending the lesion, with the exception of an occasional burning sensation. The lower eyelid was everted to such an extent that the eye could not be closed. For this reason, of course the eye was not kept sufficiently moist by the secretion of the lachrymal glands, and the cornea became dry. This condition, together with the secretion from the ulceration drying on the surface of the cornea, greatly impaired the sight of that eye. In addition to the lesion just described, there was, when he first came to me, on the bridge of his nose, extending toward the right side, a pea-sized tubercle, which made its appearance three months ago. The surface of this lesion was covered with a brownish crust, which, when removed, revealed an ulcerated base the size of a very small pea, and covered with a thin, watery secretion. The edges of this lesion were indurated and elevated. It was noticeable that the ulceration did not extend deeply, and that the induration seemed only to involve the epidermal layers of the skin.

This lesion was, in all probability, of the same character as that on the inner canthus of the eye, already described; that is, the beginning of such a lesion. I operated on this lesion a month ago, and there is no trace of the lesion to be seen at present, and in its place you see a smooth layer of cicatrical tissue. I will speak of the mode of treatment subsequently. When these lesions appear on persons past middle life, they always deserve careful consideration. An ulceration of the skin on an elderly individual, which refuses to heal up after a reasonable time, and continues to exist and give signs of degeneration of the epidermal structure should always be considered a serious affair.

These lesions come under the head of epithelioma, and there are three classes, the superficial, the deep, and the papillary; they are also known as skin cancers. The superficial variety, also called simple epithelioma, appears, as this lesion on the nose appeared, as a small papule, or tubercle, and generally involves the upper layers of the skin, as I have described in the case of the lesion on the nose. The deep variety also appears as small lesions, but the ulceration is much deeper and involves the layers of the corium and the deeper structures of the skin. The papillary variety is more or less lobulated, and extends upward from the surface. They are dentritic growths. These growths may develop from a sebaceous gland, a wart on the face, or any mother mark or nævus which you sometimes see on the faces of individuals. They have certain favorite seats, as the inner canthus of the eye, the side of the nose, the lips, the sides of the face and the forehead. The superficial variety is the most benign, as it only involves the upper layers of the skin. The lesion on the nose was of the superficial variety. The one on the inner canthus of the eye has existed such a length of time that I am afraid it involves some of the deeper structures of the skin. However, it has improved so wonderfully (the patient being now almost able to shut his eyes and his sight being restored, showing that the cornea is kept moist) that I think he may get well.

The treatment in this class of diseases should be very thorough. Caustics are generally resorted to, and among them we have the arsenical pastes, chloride of zinc, potassa, soda, and other caustic chemicals. In the eye, however, it is not feasible to use these strong caustics, as they may render the cornea opaque and produce permanent trouble. Therefore, I used the galvano-cautery. The lesion was removed with a platinum loop. I previously applied a ten per cent. solution of cocaine to the ulceration, continuing the application until anæsthesia was induced, the same being done to the lesion on the nose, so that both operations were entirely painless.

To the lesion on the side of the nose I applied caustic potash—the stick potassa. With the stick I removed all the diseased tissue. It is very easy to tell when you have it all removed, because it is very soft, and melts before the potash. When you arrive at the healthy tissue you find resistance. In using caustic potash it is always well to have a little dilute acetic acid on hand, in case the caustic goes a little too far in its action. By this means you can remedy it at once by applying the acid. The subsequent dressings of the lesions consisted of carbolized olive oil.

The lesion on his nose was operated upon a month ago, and it is, as you see, altogether healed up. In the eye are certain little suspicious spots that I will have to watch carefully, and, if necessary, operate again.

A CASE OF VARICOSE ULCER.

I have here a case which you will often see in practice, and it is the kind of a case which will often tax your patience and ingenuity as to treatment. It is one of those chronic ulcers. The point to be particularly careful about in these ulcerations is, that sometimes they are of a specific character, and therefore you have to be careful about the treatment. You see here on the upper third of the left tibial region an ulcerated surface, nearly palm sized, irregular in outline, sharply circumscribed, but the surface of the ulceration is covered with exuberant granulations. The left side presents elevations and depressions, while the right side is more even in its contour. The secretion is of a yellowish gelatinoid consistency, and here and there streaks of blood will be noticed. The lesion is excessively painful and has existed thirteen years.

In presenting this case to you, I wish more especially to call your attention to the points of differential diagnosis, especially between specific and non-specific ulcers. One of the first points to which I will call your attention is the varicose condition of the veins. The patient has been holding his leg up for sometime for your inspection, and the blood has flowed out of the veins, but you will notice as I depress the limb the blood returns and the veins become dilated. This man has an occupation that requires him to stand all the time, being employed in a cotton factory. Patients who are thus employed are specially subject to this form of disease. The standing produces a stasis of blood and a passive congestion of the cutaneous veins ensues, which, being long continued, entails an extraordinary pressure upon the tissues of the skin, and this finally assumes an inflammatory condition that continues for a time, and if neglected the tissues break down in the weakest spot, and we have the cutaneous ulcer. This ulcer is known as the varicose ulcer, and usually occurs in the middle and lower third of the tibial region. The location in this case is unusual. These ulcers are of various size, and sometimes completely encircle the limb. Sometimes they occur on one side of the body, sometimes they are symmetrical.

The next point to which I will call your attention in differentiating this disease from syphilis is the fact that this lesion is extremely painful, whereas syphilitic lesions are very rarely accompanied by any subjective symptoms, no matter how extensive they may be. The pain in this lesion is sometimes so great that the patient is not able to stand on the limb. Another point is that it has existed for fourteen years, and the ulceration is superficial, whereas a syphilitic lesion in this space of time would have produced a more extensive destruction of tissue and would have involved the deeper structures of the skin. Besides that, you would find the lesion accompanied by marked induration around its edges. The borders in this case are more or less flattened and show little evidence of induration. The last point in the differential diagnos is the absence of all specific history.

As regards the treatment of this lesion, in the first place we must produce a change in the character of the surface. It has been neglected until the granulations have piled up and given rise to the condition known as *caro luxurians* or proud flesh. As soon as the epithelial cells develop to a certain point they break down or degenerate. They do not pass on to the formation of healthy epidermal cells. Furthermore, we will have to consider in our treatment the cause of the disease, which consists in the passive congestion. For this purpose we will instruct this man how to apply a bandage to the affected limb from the toes, passing upwards with reverses to the knee, even covering the lesion. The object of the bandage is to produce pressure and give that support to the dilated vessels which the skin was not able to furnish. The bandage is put over the lesion also for the purpose of producing pressure. Upon the ulceration itself we will apply an ointment which will affect the surface. In this case I think it advisable to use a paste made up as follows :

| | |
|---|---|
| ℞—Acidi salycilici . . . . . . . . . . . . . | gr. xx. |
| Pulv. zinci oxidi . . . . . . . . . . . | ʒi. |
| Amyli . . . . . . aa | |
| Ugt. petrolati . . . . . . . . . . . . . | ℥i. |

Sig.—Apply twice daily.

Salycilic acid is supposed to produce a change in the character of the cells composing the base of ulcerations. It also has disinfectant and healing properties. The patient will furthermore be directed to wash the parts every morning with hot water and castile soap previous to the application of the ointment. This will not cure the case, and as to a prognosis, although you can give to the patient a favorable one, you must never agree to cure an ulcer in any given time. If the patient follows out directions, he will get well; it may take three months, it may take six.

---

SPONTANEOUS RUPTURE OF THE STOMACH.—Dr. J. Clarke reports two cases of spontaneous rupture of the stomach, an occurrence so rare as to render it extremely interesting, especially from a medico-legal standpoint. The two cases recorded were instances of rupture of the stomach, and not a mere perforation of the walls of that viscus. The coats of the stomach in one case were atrophied and thin, but in the other were healthy; and in neither case were the coats of the organ softened or eaten away in irregular patches by the gastric juice. In both cases the stomach was empty at the time of rupture; and in both the gastric juice acted only on the spleen, digesting off its capsule and peritoneal coat in patches, and thus allowing the escape of blood into the abdominal cavity. Dr. Clarke thinks there can be no doubt that the rupture in both took place before death, and was not due to *post mortem* softening; the seat of rupture was on the anterion wall. The mucous membrane and peritoneal covering could be traced up to the margin of the rent, and did not present any marked degree of irregularity; and there was an absence of softening, erosion, or rupture on the posterior wall of the stomach.—*The Indian Medical Gazette*, *August*, *1884*.—*Med. News*.

